# Putting performance in context: the perceived influence of environmental factors on work-based performance

**DOI:** 10.1007/s40037-015-0209-5

**Published:** 2015-09-29

**Authors:** Lynfa Stroud, Pier Bryden, Bochra Kurabi, Shiphra Ginsburg

**Affiliations:** 1 Department of Medicine, University of Toronto, Toronto, Canada; 2 Wilson Centre for Research in Education, University Health Network, University of Toronto, Toronto, Canada; 3 Department of Psychiatry, University of Toronto, Toronto, Canada

**Keywords:** Work-based assessment, Competency, Qualitative methods, Medical education

## Abstract

**Introduction:**

Context shapes behaviours yet is seldom considered when assessing competence. Our objective was to explore attending physicians’ and trainees’ perceptions of the Internal Medicine Clinical Teaching Unit (CTU) environment and how they thought contextual factors affected their performance.

**Method:**

29 individuals recently completing CTU rotations participated in nine level-specific focus groups (2 with attending physicians, 3 with senior and 2 with junior residents, and 2 with students). Participants were asked to identify environmental factors on the CTU and to describe how these factors influenced their own performance across CanMEDS roles. Discussions were analyzed using constructivist grounded theory.

**Results:**

Five major contextual factors were identified: Busyness, Multiple Hats, Other People, Educational Structures, and Hospital Resources and Policies. Busyness emerged as the most important, but all factors had a substantial perceived impact on performance. Participants felt their performance on the Manager and Scholar roles was most affected by environmental factors (mostly negatively, due to decreased efficiency and impact on learning).

**Conclusions:**

In complex workplace environments, numerous factors shape performance. These contextual factors and their impact need to be considered in observations and judgements made about performance in the workplace, as without this understanding conclusions about competency may be flawed.

## Essentials

1. Context is critical in shaping behaviours.
2. The environment is seldom considered in judgements about competency.
3. Attending physicians and learners perceive the clinical environment to have a significant impact on their performance.
4. Residents perceive their roles as managers and scholars to be most affected.
5. The extent to which the environment actually affects performance is unknown and is the subject for future study.

## Introduction

Context is critically important in shaping behaviours. Despite this, competency-based frameworks (such as the Accreditation Council for Graduate Medical Education framework in the United States [1], and its Milestones Project [2], and the CanMEDS framework in Canada [3]) view competence as something to be assessed for an individual, without explicitly considering the environment in which the performance is situated. Although there is recognition of the importance of multiple samplings of an individual’s performance *across* contexts [4], there is little focus on the actual interaction between the environment and performance in a specific context. This propensity persists although research suggests that individuals are not universally competent across contexts [5] and despite recent assertions that competence must be viewed ‘in situ’, as it is inherently bound to context [6–8].

Physician performance can vary across and within practices, changing for an individual practitioner over time and across clinical contexts [9–11]. Factors such as patient mix, patient complexity, patient volumes, and teamwork are recognized to make work-based assessment of individual physicians challenging [12]. Recently, there has been increased recognition that performance in the workplace should be viewed within the environment in which behaviour occurs [6, 13–17]. Despite this, the environment is not typically accounted for in assessments of competence of physicians. Without consideration of environmental factors and understanding their impact on performance, flawed judgements about an individual’s competence may arise [18]. Given that competency in postgraduate medicine, and during clerkship for medical students, is largely assessed in the workplace, it is essential to identify the presence of different environmental factors and understand their effect on work-based performance.

Our objective was to determine trainees’ and attending physicians’ perceptions of the environment on the Clinical Teaching Units (CTU) in Internal Medicine to understand what aspects of the environment are perceived to influence learners’ performance, which aspects of performance are most affected, and how.

## Method

### Data collection

We used constructivist grounded theory as our methodology [19]. This approach fosters the development of a new conceptual framework, where one previously did not exist, to describe a social phenomenon based on participants’ own observations and experiences. We could not find an existing conceptual framework that would capture the perceptions of participants on a CTU about how their environment may impact their performance so constructivist grounded theory seemed to be an eminently suitable approach. As explained by Charmaz, researchers’ ‘background assumptions and disciplinary perspectives alert them to look for certain possibilities and processes in their data.’ [20, p. 16]. These are known as ‘sensitizing concepts’. On our team two of the researchers, including the principal investigator and senior investigator (L.S. and S.G.), both regularly attend the CTUs and their own experiences shaped the collection and interpretation of the data. For example, we sensed that workload would be an important factor, as would physical layout of the wards, availability and helpfulness of support staff and computer systems. Thus these formed the basis of some of our open-ended questions. We maintained a reflexive approach throughout the research process, whereby we were aware of our own experiences and assumptions and took care to ensure that these did not constrain or dominate our interpretations. This process was strengthened by our two other team members who are ‘outsiders’ with respect to the CTU: one (P.B.) is a clinician in another specialty and one (B.K.) is a non-clinician.

Focus groups were used for data collection as they encourage open discussion and permit in-depth exploration of shared events, such as a CTU rotation. Because the different participant groups (attending physicians, senior residents, junior residents, and students) interact in a hierarchical relationship, complete assessments of each other, and may have unique perspectives for their cohort, separate focus groups were conducted for each level.

Attending physicians, residents, and medical students who had recently completed in-patient CTU rotations at one of the five University of Toronto urban teaching hospitals were invited to participate in level-specific focus groups. Each hospital has 4 CTUs, each comprised 1 attending physician, 1 senior (PGY2 or 3) resident, 2 or 3 junior (medicine or non-medicine PGY1) residents, and 2 senior (year 3) medical students. Beyond direct patient care, a typical day involves formal teaching at morning report and at noon rounds (didactic lecture by a faculty member) and informal teaching during team-based morning rounds on newly admitted patients and afternoon rounds; there are also daily multidisciplinary rounds. Residents are based at one hospital during each year, but may rotate through other sites for their non-CTU rotations. Faculty and students do not rotate between hospitals.

Participants were recruited by email, with responses returned anonymously to a research assistant. Participation was voluntary and all participants provided informed consent. Involvement in the study was remunerated with a small gift card to a bookstore. The University of Toronto Research Ethics Board approved this study.

Between October 2010 and July 2011, 9 focus groups with a total of 29 participants were conducted: 2 groups with attending physicians (*n* = 5), 3 groups with senior residents, (*n* = 8), 2 groups with junior residents (*n* = 6), and 2 groups with medical students (*n* = 10). An experienced research assistant (B.K.) used a scripted semi-structured interview guide (Appendix 1) to conduct each group for approximately 60 min. The guide was developed, piloted on non-participant attending physicians and senior residents, and refined based on their comments. Resident and student participants were asked to identify environmental factors on the CTU that they perceived to have affected their performance and to describe how their performance was affected across different CanMEDS roles (Box), as this framework is used in our evaluations. Attending physicians were asked about the perceived impact of environmental factors on both their learners’ performance and *on their own* performance. All levels were also asked to reflect on whether they perceived that environmental factors were taken into consideration by those assessing them. The research assistant audio-taped, anonymized, and transcribed each session.

### Data analysis

Data analysis began concurrently with data collection and used a constant comparative approach. All four investigators (L.S., P. B., B. K., and S.G.) read the initial transcripts during the open coding process and met regularly to discuss emerging themes and develop the coding scheme. The process proceeded in an iterative fashion, with early-identified themes explored in greater depth in subsequent focus groups. Analysis focused on identifying the contextual factors that were perceived by participants to have an influence on an individual’s performance while on the CTU. The CanMEDS roles that were considered to be the most affected were also identified. The team met frequently to refine and challenge the coding structure based on additional focus group transcripts. Data collection and analysis continued until theoretical saturation had been reached, the point at which further data no longer informed or challenged our emerging framework. When this was achieved the coding scheme was deemed stable. Throughout this process, the research assistant (B.K.) maintained a record of the memos generated and the evolving changes to the coding structure. NVivo qualitative data analysis software NVivo (QRS International Pty Ltd., Version9.0, 2010) was used to help organize and code the data.

## Results

**Fig. 1:**
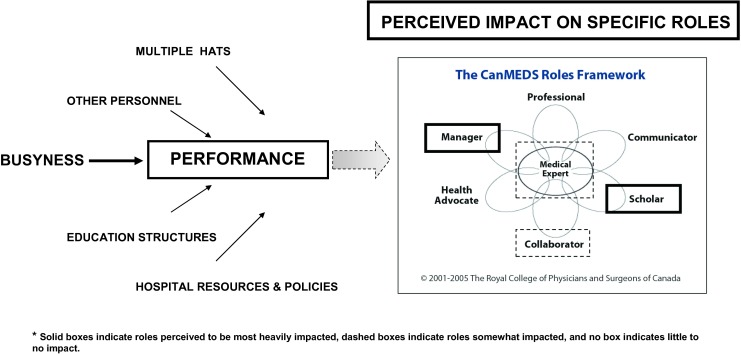
Contextual factors perceived to influence work-based performance on an Internal Medicine Clinical Teaching Unit and their impact across different CanMEDS roles. ([21])

Analysis revealed five major contextual factors, or themes, that were perceived to influence performance in the clinical setting: Busyness, Multiple Hats, Other People, Educational Structures, and Hospital Resources and Policies. Table 1 includes definitions of these factors along with further illustrative quotations. What follows is a summary of these findings related to how these factors were perceived to have influenced performance (Fig. 1; [21]).

**Table:** 

Environmental Factors	Components included in definition	Example Quote
**Busyness**	**Clinical Care of Patients:** Number of patients, turn-over of patients, medical acuity of patients psycho-social complexity of patients, cross-coverage, continuity of care	*“Workload is not just referring to your patient census, it’s referring to the severity of illness of the patients on your team, because you can certainly have a smaller census of patients but maybe a higher acuity of illness or psychosocial issues that maybe contributing to the care required.”– SR*
	**Medical Team Personnel:** Number of interns assigned to a team, house staff absences (protected time, vacation, illness), skills and background ofteam members	*“(Not) having an adequate number of house staff around so…unplanned illness, planned vacations, all of them affect workload and then affect their performance” – AP*
	**Non-clinical Work:** Amount of paperwork, clerical duties	*“It’s annoying. Faxing referrals is probably like the most cave man thing that we do around here.”–MS1 to MS2* *“Besides paging” –MS2*
**Multiple** **Hats**	**Medical Expert** Triaging sick ward patients to be seen, attending code blues, responding to ER, performing procedures	*“The majority of my colleagues are very hardworking people, and you may not be aware that they’re also doing admissions in the ER, leading the code team, teaching the medical students, reviewing patients with their staff, at that family meeting, helping their juniors with acutely ill patients and attending rounds.” – SR*
	**Non-Medical Expert** Participating in bullet rounds, leading family meetings, managing the team, teaching, attending education rounds, advocating for patients for prioritizing tests, arranging out-patient services	
	**Others Awareness of Roles** Attending physician and other health professionals lacking awareness of the amount of duties being attended to residents	*“But it’s almost like if your kind of running it smoothly then they (attending physicians) shouldn’t see it, does that make sense? Like they can’t fully appreciate what you’re doing in your day because at the end of the day you just run the list and it looks like everything is taken care of.” – SR* *“And I don’t think the nurses appreciate, particularly the complexity of a senior residents role. …The senior with the whole managerial role, I think it’s harder for the nurses to envision them in that capacity many times.” AP*
**Other** **Personnel**	**Nursing & Other Health Professionals** Availability and approachability to help with assessments, procedures, tests; patience	*“I’ve actually had really good experiences with all of the nurses and all the allied health. I think once you sort of understand what people can do and what you can do for them both ways then things work a lot better .” – JR*
	**Attending Physician** Approachability, “hands-on/off”, willingness to assist	*“I’ve worked with staff who are very hands-off and gave me enough freedom to have my own style, develop my own style and make things work. Whereas I’ve had another staff who was completely hands on and came to all the bullet rounds, watched me very closely, did things on the ward. I felt that I didn’t have as much freedom. So there was a conflict there.” – SR*
	**Patients** Interactions, requests	*“I think it’s probably more your interactions with patients and families and what they tell people. Like if they’re upset or angry, I mean that gets relayed very easily I think to your team and even to your staff. So I think it’s more the patient aspect rather than even the inter professional relationships that probably have more affect on evaluation.” – MS*
**Educational Structures**	**Rotation Transitions** Starting CTU, rotation duration, location (switching between 5 teaching hospitals), specialty (changing between sub-specialty services)	*“It’s basically like orientation on steroids, right? You may have been at X hospital one month and then you’re at Y then you’re at Z, then you’re back…it’s like starting a new job every month, with new colleagues, a new boss, your boss may change every week because your staff changes. You’re learning a new computer system, you’re learning a new hospital. So add all of that in addition to your clinical responsibilities” – SR*
	**Rotation Structures** Rotation expectations, staff change-overs and supervision, distributed call system	*“This new call system actually helps me from that perspective because the senior resident is away and you get to interact with those PGY1 and you know, you can really separate out what’s the senior residents impact on their patient care skills and what’s theirs directly.”– AP*
**Hospital Resources** **and Policies**	**Physical space and layout** Clinical spaces for seeing patients (hallway patients), space for reviewing and teaching, call rooms, lounge	*“Known affectionately as the fish bowl, umm… you know they knock or the tap on the window to (get you to) come out. I think you need to have that privacy to be able to review those cases. It’s very loud because it’s just a glass panel, you can hear the entire emergency room happening there. Sometimes I felt guilty almost doing teaching with my juniors because if they saw me doing teaching … oh, she must have time, we’ll just give her more consults, right?”– SR* *“I do admit that if someone has to do a complete abdominal exam and that’s an important part of their complaint, you do have to cut corners because you’re sitting sort of exposed in the hallway and you’re not going to expose the patient. “ – JR*
	**Ancillary Resources:** Chart availability and organization, computer access, software availability and efficiency, equipment for procedures	*“That affects your time too when you’re a) searching for a chart and b) searching for somewhere to look up labs.” – MS* *“Every ward is different, so finding the store room on each is a challenge. Finding equipment for a procedure is absolutely challenging.”–SR*
	**Policy:** 4-hour decision rule for admissions, pre-specified discharge times, geographic versus team-based other health professionals	*“Having patients assigned to allied health based on physical location is a huge hindrance to care here, rather than having the same allied health team that you meet with regularly actually take care of all of the patients that are under your care.” – SR*

*MS* Medical Student, *JR* Junior Resident, *SR* Senior Resident, *AP* Attending Physician

### Busyness

Busyness included more than just ‘workload’. Participants described that it was not just the number and turnover of patients that were important, but also the complexity of patients, the alignment of work with the available number of house staff, and the non-patient care duties. This constellation, termed, ‘Busyness’ was perceived to affect performance across many roles, mostly negatively, and was noted by all levels of participants. It especially affected the manager role, largely negatively through decreased efficiency, and impacted on residents ability to display proficiency as managers, as reported by one senior resident,

"The amount of workload definitely affects how you perform. It was quite busy. I was basically acting like an intern. I couldn’t really take the role of a senior. I was running around like an intern seeing my own patients."

Senior residents were especially vulnerable to how lack of predictability in their day also impacted their manager abilities, as succinctly described by this senior resident,

Your role is to constantly triage your priorities during the day. You can have this entire great day planned of what you want, that code pager goes off and within a minute your day has changed.

Attending physicians also acknowledged that busyness had a significant impact on resident performance and that it made the manager role all the more important, but also more demanding. As an attending physician said,

In the old days we spent a lot of time on the medical expert domain. Clinical knowledge was highly emphasized. Because we now have a high workload, the emphasis on management is crucial. Actually it’s easier to evaluate them on those domains now, because if you have a resident who’s a bad manager, you implode within seconds. You just can’t function.

Some attending physicians described that busyness can reach a threshold where it becomes challenging to assess residents abilities in the manager role, as the attending physician is much more directly involved in patient care. As an attending physician said,

I’m much more involved than I used to be, which I’m happy to do, but it makes it harder for me to judge the residents’ leadership skills because I’m essentially running the team.

However, busyness was not uniformly perceived as negative with respect to the manager role: some viewed it as a challenge to which to rise. One senior resident remarked,

Something strains the fine balance of the team- those are unpredictable things, you’ve got to roll with them, that’s where the manager role comes in.

Some individuals perceived that this might even favourably impact performance, as one medical student commented that,

Sometimes it’s a very busy night so it really pushes med students and the whole team to be on their game. So I think that it gives you an opportunity to really rise to the challenge.

Busyness also influenced performance in the scholar role, as stated by this senior resident,

Teaching on CTUs is like exercise in your life—it’s the first thing to get cancelled. If you’re too busy it’s not going to happen.

Residents also described how busyness often caused a tension between the manager and scholar roles, and they seemed to favour maximizing their performance in the manager role at the expense of the scholar role, as described by this junior resident,

As residents we sort of try to compensate for those things. Knowing that things are not flowing as efficiently as they would, maybe we skip lunch-time teaching…so that we can be done in time. Otherwise there’s no way possible.

Busyness was also perceived to have a negative impact on the medical expert role, as it seemed to increase fatigue and decrease the likelihood of doing additional reading outside of the clinical context, as one junior resident reported,

‘[you’re] more tired…you’re less likely to study…’

### Multiple hats

Participants described a tension they felt by having to wear ‘Multiple Hats’. These multiple hats referred to specific tasks, such as leading a code blue, teaching medical students, and liaising with other health professionals. The perceived effect on performance arose from trying to reconcile and achieve them all, and the belief that others, especially their attending physicians, were often unaware of these competing demands. Senior residents in particular discussed this tension and explained that they felt they had to pick and excel at some tasks that they perceived had greater influence on their performance, as they were more visible to their attending physicians and thus weighed more heavily. For example, a myriad of specific tasks were necessary every day, but if not all patients had a plan at the end of the day, they felt that their attending physicians would consider them to be inefficient, and hence poor managers. According to one senior resident:

If your staff…is not cognizant…that particularly happens more with staff who are more hands-off…and at the end of the day they just want to know what happened with your patients…not everyone’s particularly interested as to how you got there or the number of people that got you there. It’s just, ‘let’s run the list at 5 o’clock.

Attending physicians disagreed with this perception and felt that they had a good idea of the multiple hats that their residents wore, both because they felt that they understood their local contexts of practice and second because they had also been residents themselves.

### Other personnel

Other personnel, including nursing and other health professionals as well as support staff such as discharge planners and ward clerks, were usually perceived by all level of participants to enhance performance. Medical students in particular identified that these individuals had the capacity to positively influence performance on the medical expert role, as one medical student said,

I think if you have a good relationship with them, I think they can positively impact your evaluation because a lot of times they can bring things up that maybe you didn’t notice or you didn’t catch and then it helps you with problem formulations and management plans.

Residents perceived that other personnel could facilitate their performance in the manager role. Senior residents who had rotated through many different hospitals could also draw contrasts between the sites and describe specific other personnel, present at some sites but not others, who could significantly support their performance. For example, the presence of such discharge planners was perceived to positively influence efficiency.

### Educational structures

Educational structures, in particular rotating between different hospitals, were perceived to have a deleterious on performance. The manager role was felt to be most affected as transitions were felt to be detrimental to efficiency, since time was wasted navigating new landscapes. Even the attending physicians recognized this as an issue for learners, as one attending physician remarked,

That’s a huge issue because if they’re going through 5 major hospitals in the city…there’s a different way to get everything done—getting somebody to look at a diabetic foot is done by plastics at one hospital, ortho at another hospital, wound care at another hospital. It really cuts down on the efficiency.

These rotation transitions also had a perceived negative impact on both the collaborator and medical expert roles. As two senior residents said,

Maybe you couldn’t collaborate because you didn’t know who you were supposed to be collaborating with, right? [Laughs] It’s not that you didn’t want to—it’s that your rotation is 4 weeks long and it may have taken you to get to the second week to even find out that this was the person who you were supposed to be collaborating with!

You may be the world’s expert on gastroenterology but now you’re on haematology and nobody cares what you know about findings on a colonoscopy, right?

### Hospital resources and policies

Individual hospital resources and policies also influenced efficiency and again residents noted site-specific differences in some of these. For example, if the teams are not geographically aligned, and patients are bed-spaced across different wards, it was perceived to have a huge influence on performing well in the manager role. As described by one junior resident:

So the people that are bed spaced you have to go deal with a whole different team of people. And that can be (reflected) anywhere in the ITER [in-training evaluation report] …in the end it affects patient care, how long they have to stay in the hospital, and I guess how well you take care of them.

Hospital resources and policies included physical space and layout, and these were noted to have an important impact on scholarly activity. Lack of sufficient and private space for informal teaching sometimes meant that fewer sessions occurred than might otherwise have. As one senior resident said,

I’m sure that impacted my juniors because maybe they wanted to have more (teaching), but I probably did less just because it was so public and didn’t want everyone to see that

Similarly, lack of access to computer resources has an impact on trainees’ abilities to research and learn efficiently, and that affects clinical performance on the medical expert role, as this junior resident pointed out.

You just type in UpToDate™ and it makes it easy for me to have information at my fingertips. I’m now at another site that doesn’t have that. I don’t have that extra few minutes to do a literature search. That might come across in my overall compilation of certain information when I present to staff.

Specific policies also had a negative impact on development under the medical expert role. For example, a hospital policy that has a set time for a disposition decision to be made on a new emergency room consult, means that there is not time for junior residents and students to gain experience of doing a full consult. As described by one senior resident,

Most of the times the senior resident sees the patient and writes admit orders before the junior has seen [the patient], so it kind of takes away the surprise for the junior seeing them if they know what the admitting diagnosis is.

In examining the perceived impact of the 5 major factors on performance, it is worth noting what aspects of performance were *not* considered to be significantly influenced by the environment. Whereas participants discussed the manager, scholar, medical expert, and collaborator roles to variable degrees, the other three CanMEDS roles (health advocate, communicator, and professional) were discussed much less often overall.

### Extent to which influences on performance affect assessment

Residents thought that, at least to some extent, attending physicians took environmental factors into account when completing assessments of them. This was despite residents’ beliefs that attending physicians did not realize the multiple hats they wore and the number of different roles they fulfilled to adequately do their job.

Attending physicians acknowledged that the environment often constrained assessments of trainees while on CTU and that their assessments were insufficiently based on direct observation of trainees’ performance. They reported that this made them more reliant on the input of others. They also perceived that at times they, ‘cut some slack’ to trainees due to the environment, and worried that this may artificially inflate assessment scores on skills that were not directly observed. For example, one attending physician said,

It’s potentially dangerous because we’re giving higher marks; we don’t have time to observe them or we may have observed it but we just say, ‘well look, under the stress of the system we can excuse that’.

Another reported,

I forgive a lot of knowledge gaps, because I figure they’re so distracted with so many other things…

In fact, residents who were able to navigate their environments well and appear efficient were sometimes given the benefit of the doubt on other domains. One attending physician described,

I think efficient residents even with maybe less than stellar knowledge are evaluated better than non-efficient residents with better knowledge. We tend to value efficiency a lot, and if things are impeding that, the trainee looks not as competent overall.

## Discussion

Across all levels, participants felt strongly that the clinical environment had a substantial impact on performance. This supports the increasing recognition that for workplace-based performance assessments to be authentic and valid, contextual factors must be considered [6].

Attending physicians and trainees identified five key environmental factors that were perceived to impact on their performance in the clinical context on CTU. While all of these were deemed important, busyness was perceived to be the dominant factor that was most influential across performance domains. It is important to emphasize that busyness was not simply equated with workload. Instead participants talked about the alignment between clinical care demands (patient volume, acuity, complexity, and turnover; plus the non-clinical work that this generated) and medical team personnel (numbers, absences, cumulative skills; for example having one versus two medicine juniors, clerks being near the beginning or end of their rotation). This ‘balancing act’ highlights that the clinical practice environment is a highly dynamic and complex milieu; and therefore the expectation that as it changes performance should remain stable is flawed [22]. Additionally, the comments from participants about busyness, as to whether ‘their team’ had the capacity to meet demands, also echoes other recent literature in which the concept of collective competence, rather than individual competence, may be a more appropriate focus in assessing performance in the workplace [23].

Given that busyness and this ‘balancing act’ was so important, it is not entirely surprising that performance on the manager role was perceived to be most affected. For example, senior residents’ comments about how their performance was affected by unpredictability and the need to ‘constantly triage priorities’, but how this can ‘change within a minute’, speak to requisite skills in adaptability and efficiency. Difficulty with being able to respond to changes within one’s environment is on display for all to see in a challenging environment. This observability may also be why trainees sometimes chose to exhibit proficiency on the manager role, at the expense of attention to fulfilling other roles that were less observable to their attending physicians, such as attending teaching sessions for the scholar role. Another consideration for why the manager role may have been perceived to be so important is because strong performance on it may make the attending physician’s life easier, and hence over-valued [18]. This possibility was supported by comments made by some of our attending physicians and may be worthy of further investigation, as it is possible the current practice of medicine (at least in teaching hospitals) prioritizes or rewards these and related competencies over others [24].

The importance of being able to adapt to new situations was also evident in how participants talked about the impact of educational structures. We currently encourage residents to rotate through different hospitals to gain a variety of educational experiences, yet discontinuity made it challenging to perform optimally on a new service. Not only was the manager role impacted, but between *hospital* transitions made performance on the collaborator role difficult and between *rotation* transitions made performance on the medical expert role difficult. Transitions are well-recognized periods of stress and adjustment in medication education [25]. Perhaps we are ironically doing trainees an unintentional disservice with this flexibility when it comes to their performance and assessment.

In light of these findings, it is surprising that few workplace-based assessments have taken environmental factors into account. Some tools have been developed to try and characterize the clinical learning environment [26–28], yet thus far these have not been used to provide context for understanding behaviour and to frame assessments. Instead they have often been used to suggest areas for ‘improving’ the learning environment. However, the clinical practice environment is inherently complex; many aspects are not amenable to change and indeed reflect the reality of practice, yet it is within this messy setting that all work-based performance is assessed. A better understanding of the interplay between factors and performance in a specific setting is an important first step in contextualization of assessments.

The extent to which assessors take environmental factors into consideration when observing performance in the workplace is not clear. Our trainees thought that attending physicians likely considered elements of the environment in their assessments. Attending physicians did acknowledge that they considered certain environmental factors, such as busyness and educational structures, but also interestingly endorsed giving people the ‘benefit of the doubt’ and ‘cutting them some slack’, i.e. inflating assessments, on some domains (medical expert) if performance on other domains (manager) was strong. These important ‘trade-offs’ in how performance was viewed, based on environmental factors, deserves further attention.

In reflecting back on the results of our research, we can evaluate our final product using Charmaz’s four criteria for constructivist grounded theory studies [19]: credibility, originality, resonance, and usefulness. Our research meets many of the conditions characteristic of a highly credible study. Our focus groups provided us with a rich, detailed, deep understanding of participants’ perceptions of important environmental factors and their impact on individual’s performance while on the CTU. We included participants with different possible perspectives (attending physicians, residents of different levels, and students), and attained theoretical saturation. Our study findings also feature elements of originality in conceptualizing important environmental factors. For example, while we anticipated several of the environmental elements that participants discussed (such as workload and physical layout of space), many more were revealed that were not immediately self-evident. For example, the impact of educational structures, which are often implemented with good intentions to facilitate resident choice of rotation, may have unintended consequences. Even those elements that were anticipated may have greater complexity than appreciated at face value. For example, busyness did not merely equate with workload or number of patients to be seen, but also incorporated components of patient acuity, psychosocial intricacy, number of available personnel to provide coverage, competing non-clinical or clerical work, etc. Given the credibility and originality of our findings, we believe that they may resonate with others involved in CTU-based education and assessment but they might not be transferable to other clinical practice environments. We believe that the new insights offered by our findings serve as a useful stimulus for new ways to conceptualize the environment on CTU and how it may influence performance. These insights offer important considerations for work-based assessments in the CTU setting, and this is an area for future study.

There are several limitations to our study. Because focus group participation was voluntary, it was possible that those who did not participate had different views about the relevance and impact of contextual factors on performance. Some potential participants may have chosen not to volunteer due to discomfort with the focus group format, perhaps preferring individual interviews, and therefore it is possible that these individuals may have had alternate views that were not captured. Also, for feasibility reasons, two of our focus groups (1 attending physician, 1 senior resident) had only two participants and therefore were conducted as small-group interviews; this meant that some of the benefits of a larger, rich focus group discussion were perhaps missing, but this may conversely permitted a greater depth of exploration from individuals Additionally, while our individual groups represented fairly homogeneous populations (by cohort level on the same rotation), participants as a whole represented a heterogeneous group (with different levels, hospital sites, etc). Similar themes were identified across the entire group, providing evidence of triangulation, and we did reach theoretical saturation. As our goal was to explore participants’ perceptions regarding their lived experiences, we did not include direct participant observations, which may be worth considering in the future in order to generate additional insights. In addition, although the study comprised five different hospital settings, it was a single-institution study and single programme. To understand the impact of environmental factors in other specialties, further contextual information about each would be needed. The ‘issue’ of context specificity that is recognized in other areas of assessment [29], such as OSCEs, also applies to workplace-based performance. While our findings *might* apply to other internal medicine CTU settings, we would not expect them to apply broadly to surgical, ambulatory or shift-based settings. Lastly, while we explored individuals’ perceptions of how the environment impacted their performance and enquired whether they suspected this was accounted for in their assessment, we did not actually perform any analysis to determine if these perceived influences did actually affect assessments. This is a natural next step for further work in this area.

## Conclusion

In complex workplace environments, numerous factors may shape performance. Elements of the environment and their impact need to be considered in observations and judgements made about performance in the workplace, as without this understanding conclusions about competency may be flawed.

**Table 2:** CanMEDS roles and definitions

Role	Definition
**Medical Expert**	As Medical Experts, physicians integrate all of the CanMEDS roles, applying medical knowledge, clinical skills, and professional attitudes in their provision of patient centered care. Medical Expert is the central physician role in the CanMEDS framework
**Communicator**	As Communicators, physicians effectively facilitate the doctor-patient relationship and the dynamic exchanges that occur before, during, and after the medical encounter
**Collaborator**	As Collaborators, physicians effectively work within a healthcare team to achieve optimal patient care
**Manager**	As Managers, physicians are integral participants in healthcare organizations, organizing sustainable practices, making decisions about allocating resources, and contributing to the effectiveness of the healthcare system
**Health Advocate**	As Health Advocates, physicians responsibly use their expertise and influence to advance the health and well-being of individual patients, communities, and populations
**Scholar**	As Scholars, physicians demonstrate a lifelong commitment to reflective learning, as well as the creation, dissemination, application and translation of medical knowledge
**Professional**	As Professionals, physicians are committed to the health and well-being of individuals and society through ethical practice, profession-led regulation, and high personal standards of behaviour

## Appendix 1. Semi-Structured Interview Guide

1. Pre-amble about the purpose of the study
2. While you are on CTU, you work in a very complex environment. What do you think are the important elements that make up that environment?
  - If necessary probe about:
    - Physical elements (ward layouts, call rooms, emergency room, hallway patients)
    - Work structures (call schedules, hospital flow initiatives, computer systems)
    - Inter professional relationships (nurses, allied health)
    - Intra professional relationships (consulting services, ER physicians, within team, other teams)
    - Workload (patient volumes, patient complexity, patient/team sign-overs)
    - Role expectations (MD, learner, teacher, manager)
    - Absences (post-call, sickness, rounds, 1/2 days)
    - Competing demands (related to above)
    - Professional or unprofessional behaviours

3. Are certain elements of the CTU environment that you described more important than others in setting the tone of the overall environment? Or in affecting your behaviours and performance?
4. Do you think that the CTU environment that you have to work in influences your performance? Your professionalism? In what way?
5. Which elements of the CTU environment have the most impact on your performance or behaviours?
6. Every rotation, you are evaluated on ITERS, In-Training Evaluation Reports (for students: an End of Rotation Ward Evaluation; for attending physicians: a Teaching Effectiveness Score (TES)). Do you think that the CTU environment has an impact on the scores that you receive on this? If so, in what way?
7. Do you think that your attending physician (for attending physicians ‘your trainees’) takes into account the CTU environment during your rotation when they complete your evaluation? If so, how?
8. Do you think that the CTU environment affects the performance or professionalism of other members of your team? If so, how?
  - If necessary probe for thoughts about staff, seniors, juniors and students

9. Every rotation, you are asked to complete an evaluation of your attending physician (for attending physicians, ‘asked to complete an evaluation of your trainees’). Do you take into account the CTU environment when you complete this evaluation? If so, how?

## References

[1] Accreditation Council for Graduate Medical Education. ACGME Common Program Requirements. http://www.acgme.org/acgmeweb/tabid/429/ProgramandInstitutionalAccreditation/CommonProgramRequirements.aspx. Accessed 27 Oct 2014.

[2] Accreditation Council for Graduate Medical Education. Milestones. http://www.acgme.org/acgmeweb/tabid/430/ProgramandInstitutionalAccreditation/NextAccreditationSystem/Milestones.aspx. Accessed 27 Oct 2014.

[3] Royal College of Physicians and Surgeons of Canada. The CanMEDS Physician Competency Framework. http://www.royalcollege.ca/portal/page/portal/rc/canmeds. Accessed 27 Oct 2014.

[4] van der Vleuten CP, Schuwirth LW (2005). Assessing professional competence: from methods to programmes. Med Educ.

[5] Lingard L (2009). What we see and don. Adv in Health Sci Educ.

[6] Govaerts M, van der Vleuten CPM (2013). Validity in work-based assessment: expanding our horizons. Med Educ.

[7] Govaerts MJB, van der Vleuten CPM, Schuwirth LWT, Muijtjens AMM (2007). Broadening perspectives on clinical performance assessment: rethinking the nature of In-training assessment. Adv in Health Sci Educ.

[8] Kuper A, Reeves S, Albert M, Hodges BD (2007). Assessment: do we need to broaden our methodological horizons?. Med Educ.

[9] Handfield-Jones RS, Mann KV, Challis ME (2002). Linking assessment to learning: a new route to quality assurance in medical practice. Med Educ.

[10] Durning SJ, Artino AR, Boulet JR, Dorrance K, Van der Vleuten CPM, Schuwirth LWT (2012). The impact of selected contextual factors on experts. Adv Health Sci Educ Theory Pract.

[11] Ginsburg S, Bernabeo E, Ross KM, Holmboe ES (2012). It depends. Acad Med.

[12] Norcini J (2005). Current perspectives in assessment: the assessment of performance at work. Med Educ.

[13] Lurie SJ, Mooney CJ, Lyness JM (2009). Measurement of the general competencies of the Accreditation Council for Graduate Medical Education: a systematic review. Acad Med.

[14] Hoff TJ, Pohl H, Bartfield J (2004). Creating a learning environment to produce competent residents: the roles of culture and context. Acad Med.

[15] Ten Cate O, Snell L, Carraccio C (2010). Medical competence: the interplay between individual ability and the health care environment. Med Teach.

[16] Crossley J, Jolly B (2012). Making sense of work-based assessment: ask the right questions, in the right way, of the right people. Med Educ.

[17] Lurie SJ (2012). History and practice of competency-based assessment. Med Educ.

[18] Ginsburg S, McIlroy J, Oulanova O, Eva K, Regehr G (2010). Towards authentic clinical evaluation: pitfalls in the pursuit of competencies. Acad Med.

[19] Charmaz K (2009). Constructing Grounded Theory: a practical guide through qualitative analysis.

[20] Charmaz K (2009). Constructing Grounded Theory: a practical guide through qualitative analysis.

[21] Frank JR, Danoff D (2007). The CanMEDS initiative. Med Teach.

[22] Sturman MC, Cashen LH, Ceramie RA (2005). The impact of job complexity and performance measurement on the temporal consistency, stability, and test-retest reliability of employee job performance ratings. J Appl Psychol.

[23] Lingard L., Hodges BD, Lingard L (2012). Rethinking competence in context of teamwork. The question of competence: reconsidering medical education in the Twenty-First Century.

[24] Ginsburg S, Gold W, Cavalcanti RB, Kurabi B, McDonald-Blumer H (2011). Competencies. Acad Med.

[25] Bernabeo EC, Holtman MC, Ginsburg S, Rosenbaum JR, Holmboe ES (2011). Lost in transition: the experience and impact of frequent changes in the inpatient learning environment. Acad Med.

[26] Roff S, McAleer S, Skinner A (2005). Development and validation of an instrument to measure the postgraduate clinical learning and teaching educational environment for hospital-based junior doctors in the UK. Med Teach.

[27] Boor K, van der Vleuten C, Teunissen P, Scherpbier A, Scheele F (2011). Development and analysis of D-RECT, an instrument measuring residents. Med Teach.

[28] Soemantri D, Herrera C, Riquelme A (2010). Measuring the educational environment in health professions studies: a systematic review. Med Teach.

[29] Van der Vleuten CPM (2014). When I say… context specificity. Med Educ.

